# Long-term excessive salt consumption alters villous and crypt morphology and the protein expression of uroguanylin, TRPV6 and PMCA_1b_ in the rat small intestine

**DOI:** 10.1371/journal.pone.0317415

**Published:** 2025-01-16

**Authors:** Natchayaporn Thonapan, Kannikar Wongdee, Sirion Aksornthong, Jarinthorn Teerapornpuntakit, Wacharaporn Tiyasatkulkovit, Nattapon Panupinthu, Narattaphol Charoenphandhu

**Affiliations:** 1 Graduate Program in Molecular Medicine, Faculty of Science, Mahidol University, Bangkok, Thailand; 2 Center of Calcium and Bone Research (COCAB), Faculty of Science, Mahidol University, Bangkok, Thailand; 3 Faculty of Allied Health Sciences, Burapha University, Saen Suk, Chonburi, Thailand; 4 Department of Physiology, Faculty of Science, Mahidol University, Bangkok, Thailand; 5 Physiology Division, Preclinical Sciences, Faculty of Medicine, Thammasat University, Pathum Thani, Thailand; 6 Department of Biology, Faculty of Science, Chulalongkorn University, Bangkok, Thailand; 7 Institute of Molecular Biosciences, Mahidol University, Nakhon Pathom, Thailand; 8 The Academy of Science, The Royal Society of Thailand, Dusit, Bangkok, Thailand; University of Hawai’i at Manoa, UNITED STATES OF AMERICA

## Abstract

Although long-term high dietary sodium consumption often aggravates hypertension and bone loss, sodium in the intestinal lumen has been known to promote absorption of nutrients and other ions, e.g., glucose and calcium. However, whether high-salt diet (HSD) altered mucosal morphology, villous cell turnover and calcium transporter expression remained elusive. Herein, rats were treated with HSD containing 8% wt/wt NaCl for up to 5 months. HSD rats exhibited a marked increase in sodium intake with high fecal and urinary sodium excretion, as compared to the control group treated with normal diet. Intestinal histomorphometry revealed increasing of crypt depth and villous height in 3- and 4-month HSD groups, respectively, consistent with larger mucosal-to-serosal amplification ratio that reflected an increased surface area for nutrient absorption. The signals of Ki-67-positive cells was enhanced in the crypts as visualized by multiphoton fluorescence microscopy, whereas the TUNEL-positive cells were decreased in the villi of HSD, suggesting greater crypt cell proliferation and a reduction of villous cell apoptosis. Confocal microscopy showed higher expression of TRPV6 protein in the villous tip of HSD, while PMCA_1_ expression was increased in villous tip and crypt areas. The percentage of cells with highly expressed uroguanylin—an endogenous intestinal natriuretic peptide—was significantly higher in HSD group. In conclusion, HSD profoundly changed the intestinal morphology and turnover of epithelial cell, increased the expression of calcium transporters and uroguanylin. Our findings reflect pathophysiological adaptations in the intestine, which might be another target organ for drug discovery against HSD-induced osteopathy in the future.

## Introduction

High salt consumption mostly from processed food and sauces—e.g., ham, sausages, canned food, and cheese—often aggravates non-communicable diseases (NCD), particularly hypertension, cardiovascular disease, stroke and diabetes mellitus [1–3]. Recently, we have reported that high salt consumption in rats increased blood pressure and impaired bone microstructure, which may, in turn, contribute to a higher risk of osteoporosis and fragility fractures [4–7]. Specifically, as demonstrated by the calcium balance study, long-term (5 months) high-salt diet (HSD) increased fecal calcium loss and decreased the fractional calcium absorption [7], presumably as a result of luminal hyperosmolarity that trapped water and certain water-soluble minerals (e.g., calcium and magnesium) within the lumen [8]. Therefore, it was hypothesized that the intestinal cells themselves needed to trigger several compensatory responses to enhance absorptive capability, such as altering mucosal morphology (e.g., villous adaptation and crypt cell turnover), enterocyte proliferation and apoptosis [9], and upregulating the expression of calcium transporter proteins under HSD conditions.

Generally, the small intestine is a major site for Na^+^ absorption. There are two major pathways linked to the apical Na^+^ entry of intestinal epithelial cells, i.e., (*i*) nutrient-coupled Na^+^ absorption (e.g., Na^+^-dependent sugar transport by Na^+^ or glucose cotransporter 1 (SGLT1)], and (*ii*) electroneutral NaCl absorption mediated by Na^+^/H^+^ exchangers (e.g., NHE2, NHE3, NHE8) and Cl^−^/HCO_3_^−^ exchangers. The predominant route for basolateral Na^+^ extrusion in both small and large intestine is via the Na^+^/K^+^-ATPase (NKA) [10, 11]. Although it is unclear how an increase in cellular Na^+^ transport as seen with HSD intake affects intestinal morphology, chronic exposure to high intraluminal concentrations of Na^+^ may lead to alterations in body sodium balance and potentially changes in intestinal mucosal characteristics [12, 13]. For instance, rats given water containing 1% NaCl for 4 weeks exhibited a decrease in the intestinal absorption of Na^+^, Cl^−^, and water in the absence of change in blood or extracellular fluid volume [14]. In the small intestine of rats treated with water containing 1.5% NaCl for 7 days, there was a ~40% downregulation of NHE3—an apical membrane transporter crucial for transepithelial Na^+^ uptake and H^+^ efflux into the lumen—and ~55% reduction of the electroneutral Na^+^/HCO_3_^−^ cotransporter (NBCn2), thereby decreasing Na^+^ and Cl^−^absorption capacity and restricting salt uptake into the body [15]. Since NHE3 knockout mice also exhibited a decrease in the duodenal calcium absorption probably due to a reduction in the solvent drag-induced calcium transport [16], it was, therefore, possible that high salt-induced reduction in NHE3 activity at the apical membrane could also reduce the fractional calcium absorption. Nevertheless, whether HSD altered the expression of NKA, which is essential for basolateral Na^+^ extrusion and salt uptake into the circulation, remained unclear.

On the other hand, HSD may trigger compensatory protective mechanisms to help prevent salt excess. It has been reported to induce the intestinal production of uroguanylin (UGN), an endogenous intestinal natriuretic peptide [17, 18] that activates the guanylate cyclase-C receptor in enterocytes and renal tubular cells, thereby inhibiting intestinal Na^+^ uptake and enhancing urinary Na^+^ excretion [19]. Meanwhile, other intestinal mucosal cells, such as goblet cells, also contribute to the regulation of luminal osmolarity by secreting water to dilute salt and mucin to create a barrier for Na^+^ uptake [20]. Until now, it was not known whether HSD could modulate UGN expression and goblet cell populations in the intestinal villi.

Regarding the effect of HSD on calcium metabolism, besides being a factor that induced fecal calcium loss, HSD was previously reported to enhance urinary calcium excretion and induce hypocalcemia, the latter of which consequently activated the secretion of parathyroid hormone (PTH) and 1,25-dihydroxyvitamin D_3_ [1,25(OH)_2_D_3_] [21, 22]. Generally, 1,25(OH)_2_D_3_ was capable of upregulating the expression of calcium transporters, e.g., transient receptor potential cation channel subfamily V member 6 (TRPV6) and plasma membrane Ca^2+^-ATPase 1b (PMCA_1b_) [23–26]. Both proteins were abundantly expressed in the enterocytes and were responsible for apical calcium uptake and basolateral calcium extrusion, respectively [27]. We thus hypothesized that the small intestinal mucosa may undergo some morphological adaptations or changes in transporter expression as compensatory responses to long-term HSD intake in order to maintain intestinal calcium uptake.

Therefore, the objectives of the present study were (*i*) to perform a histomorphometric analysis to reveal intestinal mucosal adaptations during long-term HSD treatment, including cell proliferation and apoptosis, and (*ii*) to determine the protein expression of calcium transporters in the intestinal mucosa of HSD-treated rats.

## Materials and methods

### Animals

Eight-week-old male Sprague Dawley rats (weighing 200–220 g) were obtained from the Nomura Siam International Co. Ltd. (Bangkok, Thailand). Rats were housed in stainless steel cages (20–22°C, 50–60% relative humidity under 12/12 h light–dark cycle) at Central Animal Facility, Faculty of Science, Mahidol University accredited by Association for Assessment and Accreditation of Laboratory Animal Care International (AAALAC). All rats were acclimatized for 7 days and spent an additional 7 days undergoing baseline blood pressure monitoring. They were fed a standard normal diet (Perfect Companion Group Co., Ltd., Bangkok, Thailand) and given reverse osmosis (RO) water ad libitum. The method of sacrifice was an intraperitoneal injection of 100 mg/kg body weight of sodium pentobarbitone (Abbott Laboratories, North Chicago, IL, USA). During blood collection, the method of anesthesia/analgesia involved inhalation of 5% isoflurane (induction), followed by maintenance with 3–4% isoflurane (Abbott Laboratories). To alleviating animal suffering, the rats were kept warm with a blanket and heat lamp in a low-stress tranquil environment. The animal protocol has been approved by the Institutional Animal Care and Use Committee (IACUC), Faculty of Science, Mahidol University (Protocol no. MUSC60-045-395 and MUSC63-023-531). Our studies related to animals and specimens (e.g., blood and tissues) were complied with ARRIVE guideline (https://arriveguidelines.org/ARRIVE-guidelines/).

### Experimental design

At 10 weeks of age, rats were randomly divided into two groups i.e., that fed normal salt diet (0.8% wt/wt NaCl; Control) as control group or high-salt diet (8% wt/wt NaCl; HSD) [28]. Control or HSD were given daily ad libitum up to 5 months [6]. Sodium balance study was performed monthly. Rats were finally euthanized at the designated ages of 1, 2, 3, and 4 months. Bone formation marker (N-terminal propeptide of type 1 procollagen; P1NP; cat. no. AC-33F1; Immunodiagnostic Systems, Bolden, UK), and resorption marker (C-terminal telopeptide of type 1 collagen; CTX-1; cat. no. AC-06F1; Immunodiagnostic Systems) were determined by commercial ELISA kits according to the manufacturer’s instruction. Duodenal tissues were collected for histological staining, histomorphometric analysis and protein expression study.

### Sodium balance study

The rats were placed in metabolic cages for a three-day sodium balance study, with daily monitoring of food and water intake, urine volume, fecal weight, and body weight. Food pellets, feces, and urine samples were collected for sodium content analysis at the end of the monitoring period. Sample preparation and sodium content assessment were adapted from a previous study by Suntornsaratoon et al. [29]. Dry food was ashed at 800°C overnight in a muffle furnace, and the ash weight was recorded. One milligram of food ash was dissolved in acid mixture containing 7 mL of concentrated HNO_3_ and 1 mL of H_2_O_2_ using microwave digestion. The digested samples were diluted to a final volume of 25 mL with deionized water and analyzed for sodium content using an atomic absorption spectrophotometer. Sodium intake was calculated using Eq 1.


$${Na}^{+}\;intake(mg/day)={Na}^{+}content\;in\;dry\;food(mg/mg)\times Average\;daily\;dry\;food\;consumption(mg/day)$$ (1)

The percentage of Na^+^ retention was calculated to represent the amount of Na^+^ retained by the body (Eq 2), accounting for salt intake and sodium excretion through urine and feces (total Na^+^ excretion).


$${Na}^{+}\;retention(\%)=\frac{\;{Na}^{+}\;intake\;\left(mg\right)-Total\;{Na}^{+}\;excretion(mg)}{{Na}^{+}\;intake(mg)}\times 100$$ (2)

### Tissue preparation for histological study

Distal duodenal tissues (1–2 cm in length) were dissected from each animal (5–8 animals per group), cleaned of luminal contents with phosphate-buffered saline (PBS), and fixed overnight in 4% formaldehyde solution at 4 C. The fixed tissues were then dehydrated by serial ethanol immersion and cleared with xylene. Tissues were embedded in 2–3 separate paraffin blocks and cut longitudinally into 5-μm-thick sections by a rotary microtome (model RM2255; Leica Biosystems, Nussloch, Germany) with high-alloyed stainless steel low-profile disposable blades (model 819; Leica Biosystems). To ensure optimal sectioning quality, blades producing unsmooth sections or tissue distortion were immediately replaced. Tissue sections (3–5 sections per paraffin block, depending on tissue orientation within the blocks and section quality) were randomly selected for staining with hematoxylin & eosin (H&E) [30] or Alcian blue (pH 2.5)/nuclear fast red [31] for morphometric and goblet cell analysis, respectively. Approximately 30–60 images for intestinal morphometry and 8–10 images for goblet cell analysis were captured using a light microscope (model BX51TRF; Olympus, Tokyo, Japan) at 100× magnification.

### Intestinal histomorphometric analysis

Villous height (VH), villous width (VW), crypt depth (CD), and crypt width (CW) were measured from 40 villi and crypts of 3–5 duodenal sections per animal. The morphometric measurements were performed as previously described by Kisielinski et al. [32]. In brief, VH was determined by the length from the top of the villus to the crypt opening. VW was measured from the edge of the villous brush border membrane to the opposite side of its epithelial border. CD was defined as the distance from the crypt bottom to the invagination between two villi. CW was measured from the base of cell lining in the transit amplification zone of the crypt to the opposite side of the cell base. The calculation of the mucosal-to-serosal amplification ratio (*M*), which determined the histological adaptation of the mucosal absorptive surface, was performed according to Eq 3, as previously described by Kisielinski et al. [32].


$$\text{M}=\frac{\left(\text{VW}\times \text{VH}\right)+{\left(\frac{\text{VW}}{2}+\frac{\text{CW}}{2}\right)}^{2}-{\left(\frac{\text{VW}}{2}\right)}^{2}}{{\left(\frac{\text{VW}}{2}+\frac{\text{CW}}{2}\right)}^{2}}$$ (3)

The positive blue color (alcianophilic blue) of goblet cells per villus was counted from 10 villi of 3–5 duodenal sections per animal. All measurements were analyzed by Nikon NIS-Element BR version 4.0 (Nikon, Tokyo, Japan).

### Protein expression analyses by using multiphoton microscopy and confocal laser-scanning microscopy

The duodenal sections were deparaffinized with xylene and rehydrated by serial ethanol immersion. Optimal antigen retrieval processes were then applied before immunostaining. Briefly, sodium citrate buffer containing 10 mM sodium citrate and 0.05% Tween 20 (pH 6.0) was applied at 95°C for 30 min to unmask Ki-67 antigen, and proteinase K solution (0.01 mg/mL proteinase K, 50 mM Tris-HCl pH 8.0, and 5 mM EDTA) was applied at 37°C for 30 min to unmask target antigens, e.g., NKA and TRPV6. Tris/EDTA buffer (10 mM Tris base, 1 mM EDTA solution, 0.05% Tween 20, pH 9.0) was applied at 95°C for 30 min to retrieve PMCA_1_ and UGN antigens. Non-specific binding was blocked for 2 h with a blocking solution (4% bovine serum albumin, 5% normal serum, and 0.1% Tween-20 in PBS). The effectiveness of the blocking was confirmed by the absence of signal in the negative control section, in which the primary antibody was not applied.

Thereafter, sections were incubated overnight with 1:100 rabbit anti-Ki-67 primary antibody (ab16667; Abcam, Waltham, MA, USA), 1:100 rabbit anti-NKA primary antibody (ab76020; Abcam), 1:100 rabbit anti-TRPV6 primary antibody (SC-28763; Santa Cruz Biotechnology, Dallas, TX, USA), 1:500 rabbit anti-PMCA_1_ (ab190355; Abcam), and 1:250 rabbit anti-UGN primary antibody (18113-1-AP; Proteintech, Chicago, IL, USA). The negative control sections were incubated only with the blocking solution (without primary antibody). The sections were then washed in PBS containing 0.1% Tween-20. Ki-67 was detected by incubating with 1:500 goat anti-rabbit IgG conjugated DyLight 488 (DI-1488; Vector Laboratories, Burlingame, CA, USA), whereas NKA, TRPV6, PMCA_1_ and UGN were detected by incubating at room temperature for 1 h in a dark chamber with 1:500 goat anti-rabbit IgG conjugated with DyLight 594 (DI-1594; Vector Laboratories). Nuclei were stained with 4’,6-diamidino-2-phenylindole (DAPI) in antifade mounting media (S36964; ThermoFisher Scientific, Waltham, MA, USA). Photomicrographs of Ki-67, TRPV6, PMCA_1_ and UGN were captured by a multiphoton microscope (model Leica SP8 DIVE; Leica Microsystems, Wetzlar, Germany) at 409–450 nm excitation for DAPI, 380–398 nm excitation for DyLight 488, and 630–720 nm excitation for DyLight 594 using HC PL IRAPO 25× water objective lens with a numerical aperture of 1.00, zoom ratio of 2 for Ki-67, zoom ratio of 3 for TRPV6 and PMCA_1_, and zoom ratio of 0.75 for UGN detections. The low-magnification representative images of TRPV6 and PMCA_1_ were captured by using Zeiss LSM800 confocal laser-scanning microscope (Carl Zeiss AG, Germany), and representative images of NKA were captured by using FV10i-DOC confocal laser-scanning microscope (Olympus, Tokyo, Japan).

Regarding the quantitative determination of immunofluorescent signals, the number of Ki-67 positive cells per crypt and percentage of Ki-67 positive cells per crypt were determined at 10 crypts per animal. Crypts were identified as intact, elongated tubular structures located beneath a single villus, extending from the base to the bottom of the epithelial layer. Only well-oriented crypts without damage, marked distortion or artifacts were included. To ensure consistency in Ki-67-positive cell assessment, immunostaining was optimized, images were obtained at a constant magnification. Crypts were selected randomly, and only cells with clear nuclear Ki-67 and DAPI signals were counted.

The immunofluorescent signal intensities of NKA, TRPV6, and PMCA_1_ were measured within the designated regions of interest (ROI) along the intact structure of villus–crypt axis, i.e., crypt base (CB), crypt (C), crypt–villus junction (CVJ), villus (VL), and villous tip (VT) as described by Vincent et al. [33], all of which were randomly obtained from 6–8 different fields per animal. The mean average intensities of NKA and PMCA_1_ were measured at the basolateral membrane of intestinal epithelial cells, whereas that of TRPV6 was measured at the apical membrane of the epithelial cells. The ratio of the number of highly UGN-positive cells per total cells and the fold difference of mean average intensity of UGN between ND and HSD groups were obtained from 10 villi per animal. Image analyses were performed by Nikon NIS-Element BR version 4.0 (Nikon).

### Determination of apoptotic cells by terminal deoxynucleotidyl transferase dUTP nick end labeling (TUNEL) assay

TUNEL assay was used to visualize apoptosis in the intestinal mucosa. After being deparaffinized and rehydrated, the duodenal sections were permeabilized with 0.1% Triton X-100 in PBS for 8 min and washed in PBS. The sections were then incubated with fluorescein (FITC)-labeled TUNEL reaction (catalog no. 11684795910; Roche Diagnostics GmbH, Mannheim, Germany) at 37°C under dark and humidified atmosphere for 1 h. The negative control sections were incubated in buffer without enzyme. Thereafter, the sections were washed with PBS and mounted with DAPI-containing antifade mounting media (S36964; ThermoFisher Scientific). Images were captured by a Leica SP8 DIVE multiphoton fluorescence microscope (Leica Microsystems) at 409–450 nm excitation for DAPI and 380–398 nm excitation for DyLight 488 using HC PL IRAPO 25× water objective lens with numerical aperture of 1.00 and zoom ratio of 0.75. Apoptotic cells were assessed by counting number of TUNEL-positive nuclei per villus, and calculating percentage of TUNEL-positive nuclei per villus from 10 villi per animal using NIS-Element BR version 4.0 (Nikon).

### Statistical analysis

The results are expressed as mean ± SE. An *F*-test was used to check whether the variances of two or more populations were equal. Comparisons between two groups with normal distribution were made using an unpaired Student’s *t*-test. Multiple comparisons were performed to assess the expression levels of NKA, TRPV6, and PMCA_1_ along the villus-crypt axis using a one-way analysis of variance (ANOVA), and differences between pairs of means were analyzed using Tukey’s post-test. All statistical analyses were performed using GraphPad Prism 9 (GraphPad Software Inc., San Diego, CA, USA). The level of significance was set at *P*-value less than 0.05.

## Results

**Fig 1:**
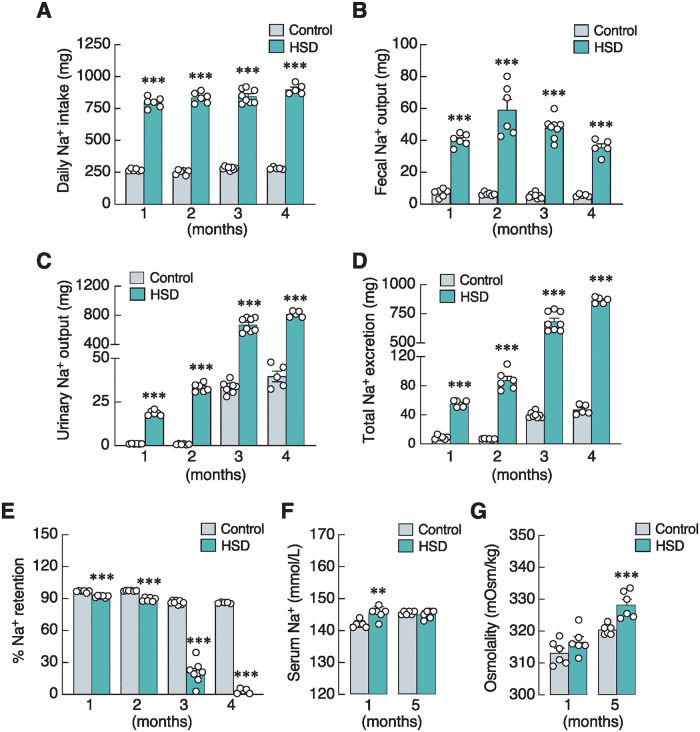
Effects of high-salt diet (HSD) on body sodium balance. (A) Daily sodium intake, (B) fecal sodium excretion, (C) urinary sodium excretion, (D) total sodium excretion, (E) percent of sodium retention of rats fed with HSD for 1–4 months. (F) Serum sodium level, and (G) serum osmolarity in rats fed HSD for 1 and 5 months (n = 5–8/group; unpaired Student’s *t*-test; **P < 0.01, ***P < 0.001 vs. age-matched control group).

As shown in Fig 1, the results from sodium balance study showed that high-salt diet treatment caused an increase in daily Na^+^ intake by ~3.5-fold, and was significantly associated with greater fecal Na^+^ output, urinary Na^+^ output, and total Na^+^ excretion in HSD when compared with the age-matched control groups. Thus, the reduction in percent Na^+^ retention in HSD group dependent on treatment duration (Fig 1E). Two-way ANOVA showed an interaction between HSD and treatment duration (S1 Table) demonstrating that prolonged HSD exposure (3–4 months) led to a more marked reduction in Na^+^ retention, suggesting that the kidney probably activated a compensatory response to enhance Na^+^ excretion, consistent with high urinary Na^+^ output up to ~800 mg/day in 3- and 4-month HSD groups (Fig 1C). An elevated serum Na^+^ level was observed in 1-month HSD group, but not 5-month group (Fig 1F), whereas serum osmolarity was higher in 5-month group, but not 1-month HSD group (Fig 1G).

**Fig 2:**
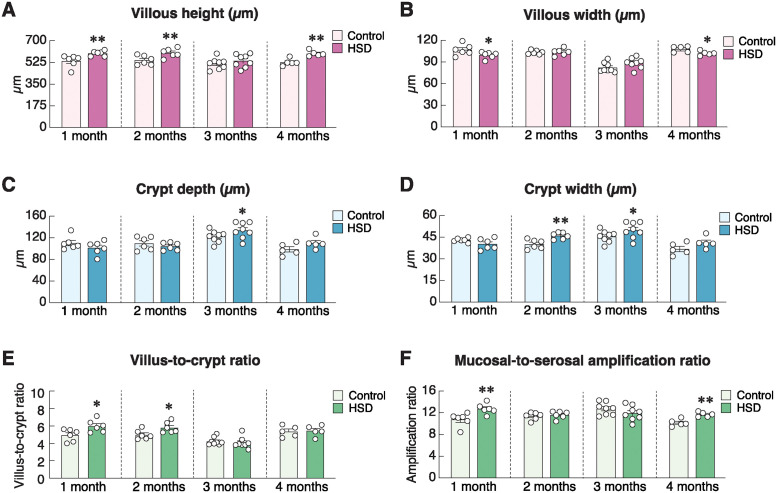
Effects of high-salt diet (HSD) on morphological structures and absorptive surface area of the rat duodenum. (A) Villous height, (B) villous width, (C) crypt depth, (D) crypt width, and (E) villous-to-crypt height ratio, (F) mucosal-to-serosal amplification ratio, i.e., an indicator of the enlarged absorptive surface area, in rats fed HSD for 1–4 months (n = 5–8/group; unpaired Student’s *t*-test; *P < 0.05, **P < 0.01 vs. age-matched control group).

**Fig 3:**
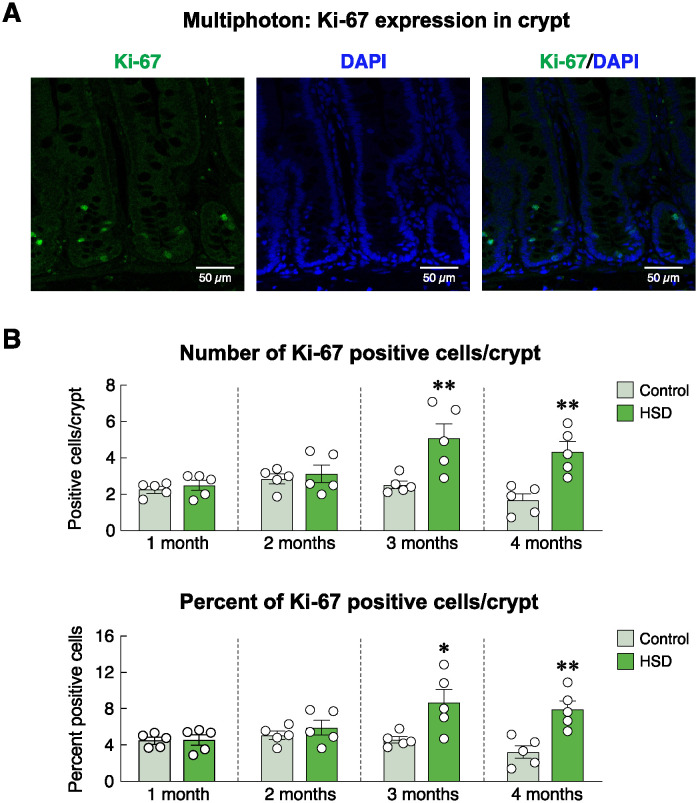
Effects of high-salt diet (HSD) on proliferation of duodenal crypt cells as indicated by Ki-67 immunofluorescent staining. (A) Representative immunofluorescent photomicrographs obtained from a multiphoton microscope. Green fluorescent signals indicated the Ki-67-positive (proliferating) cells. Blue signals of DAPI represent nuclei of crypt cells (scale bars, 50 μm). (B) Number and percentage of Ki-67-positive cells/crypt in rats fed HSD for 1–4 months (n = 5/group; unpaired Student’s *t-*test; *P < 0.05, **P < 0.01 vs. age-matched control group).

**Fig 4:**
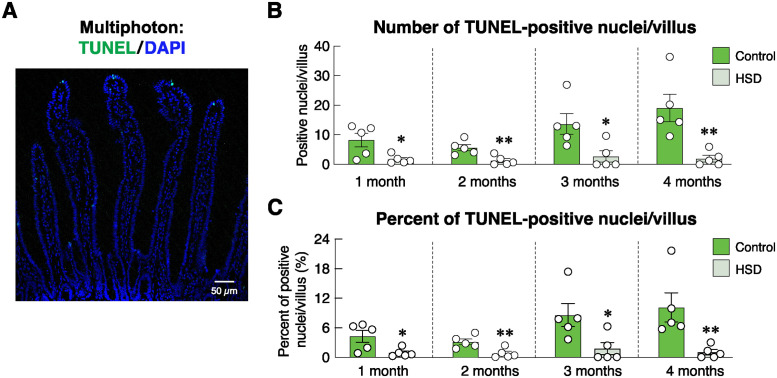
Effects of high-salt diet (HSD) on apoptosis of villous epithelial cells in the rat duodenum. (A) Representative photomicrograph of TUNEL assay obtained from multiphoton microscope. Positive green fluorescent signals of TUNEL reaction represent apoptotic cells, whereas blue signals of DAPI represent nuclei of the villous epithelial cells (scale bar, 50 μm). (B) Number of TUNEL-positive cells/villus, and (C) percentage of TUNEL-positive nuclei/villus in rats fed HSD for 1–4 months (n = 5/group; unpaired Student’s *t-*test, *P < 0.05, **P < 0.01 vs. age-matched control group).

Regarding the intestinal mucosal adaptation after HSD treatment (Fig 2A–2D), our histomorphometric study revealed that HSD increased the duodenal villous height at 1, 2 and 4 months, while decreasing villous width at 1 and 4 months. Duodenal crypts appeared to widen and increase in height at 3 months. The villous/crypt height ratio was greater in the 1- and 2-month HSD groups, but not in the 3- or 4-month HSD groups (Fig 2E). These dynamic morphological changes reflected the enhanced absorptive capacity of the mucosa, as indicated by a greater mucosal-to-serosal amplification ratio at 1 and 4 months of HSD treatment (Fig 2F). Since the greater mucosal-to-serosal amplification ratio was a proxy indicator of an increased surface area for nutrient and mineral absorption [34], we further hypothesized that crypt cells became highly proliferative and/or apoptosis in the villous area declined. Immunofluorescent analysis of Ki-67 expression—a cell proliferation marker [35]—revealed that HSD induced a significant increase in crypt cell proliferation at 3 and 4 months, but not 1- and 2-month post-HSD treatment (Fig 3). Representative photomicrographs captured by using a multiphoton fluorescence microscope showed positive green signals of Ki-67-positive proliferating cells, particularly in the crypt region (Fig 3A). Furthermore, TUNEL assay confirmed that TUNEL-positive apoptotic cells and percent TUNEL-positive nuclei per villus were significantly lower in 1-, 2-, 3- and 4-month HSD group than the age-matched ND control group (Fig 4).

**Fig 5:**
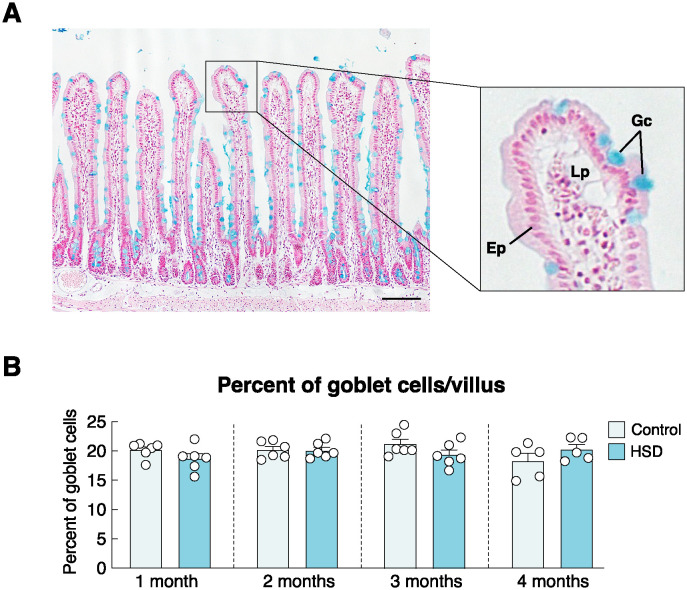
Effects of high-salt diet (HSD) on changes of goblet cell numbers in duodenal villi. (A) Representative photomicrograph of duodenal villi stained with Alcian blue. Bluish-green staining signals of goblet cells (Gc) are distributed along the villous mucosa containing epithelial cells (Ep) and in the crypts (scale bar, 100 μm). Lp, lamina propria. (B) Percentage of goblet cells/villus in rats fed HSD for 1–4 months (n = 5–6/group; unpaired Student’s *t*-test).

Since goblet cells were responsible for the production of mucin onto the mucosal surface [36], their proliferation might be a mechanism to help increase the mucous barrier against luminal high salt or impede Na^+^ diffusion. Hence, we determined the effect of long-term HSD on the alteration of goblet cells using Alcian blue staining. The results showed bluish-green-positive for Alcian blue staining in goblet cells lining along the villous epithelium as well as in the intestinal crypts. Nevertheless, similar goblet cell numbers per villus in the HSD and control groups indicated that HSD did not affect the duodenal goblet cell population (Fig 5).

**Fig 6:**
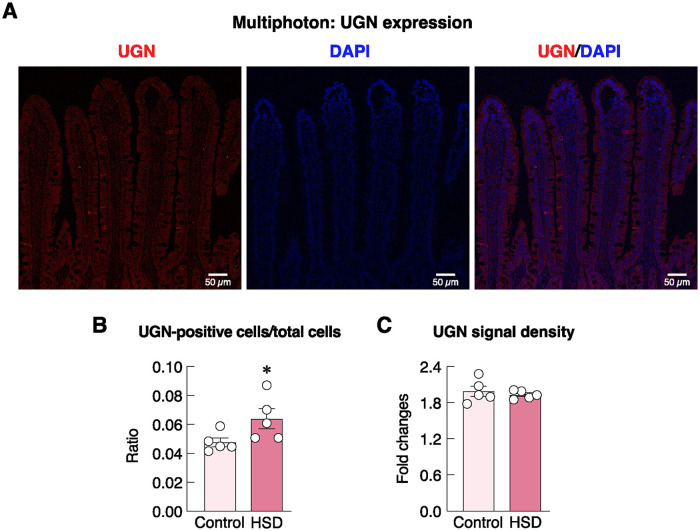
Expression of uroguanylin (UGN) in the duodenal villi. (A) Representative immunofluorescent photomicrographs showing UGN distribution in the duodenal villi as visualized by a multiphoton microscope. The bright red fluorescent signals of UGN-expressing cells are distributed in the villous epithelial cells, especially at the villous portion. Nuclei are stained with DAPI (blue signals). Scale bars, 50 μm. (B) Ratio of UGN-positive cells/total cells, (C) fold changes of UGN signal density of the highly UGN-positive cells per normal UGN-positive cells in rats fed HSD for 3 months (n = 5/group; unpaired Student’s *t*-test; *P < 0.05 vs. age-matched control group).

**Fig 7:**
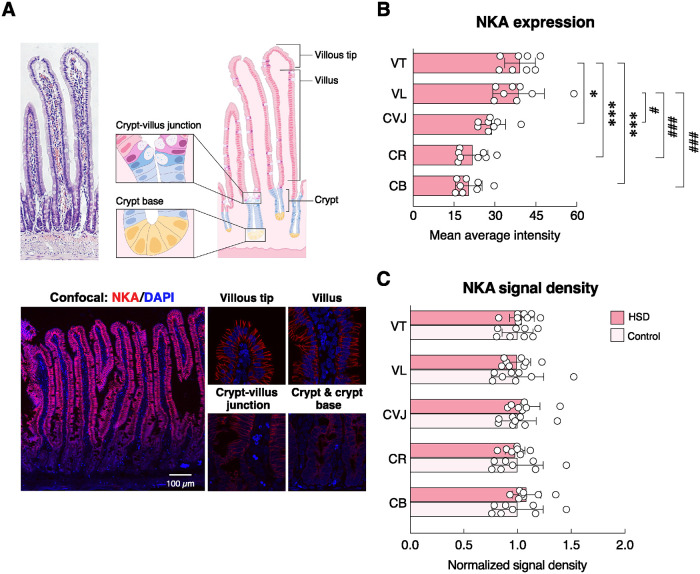
Expression of Na^+^/K^+^ ATPase (NKA) protein and the effects of 3-month high-salt diet (HSD) ingestion on NKA expression along the crypt-villus axis in rat duodenum. (A) An artwork demonstrates different parts of the duodenal villus used for quantification of immunofluorescent signals, i.e., villous tip (VT), villus (VL), crypt-villus junction (CVJ), crypt (CR), crypt base (CB). Representative fluorescent photomicrographs captured by a confocal laser-scanning microscope (150× and 1200×; Olympus model FV10i-DOC) show NKA protein expression (red) in the basolateral membrane of 22-week-old rat duodenum [blue, nuclei stained with 4’,6–diamidino–2–phenylindole (DAPI)]. (B) Mean average intensity of NKA protein expression from VT to CB in 22-week-old rat duodenum. *P < 0.05; ***P < 0.001 vs. expression level at the VT; ^#^*P* < 0.05, ^###^*P* < 0.001 vs. expression level at the VL. (n = 8; one-way ANOVA with Tukey’s multiple comparisons test). (C) Normalized signal density of NKA protein along different parts of the duodenal villus in rats fed HSD for 3 months. Expression levels of age-matched control groups are normalized to 1 (n = 8/group; unpaired Student’s *t*-test).

**Fig 8:**
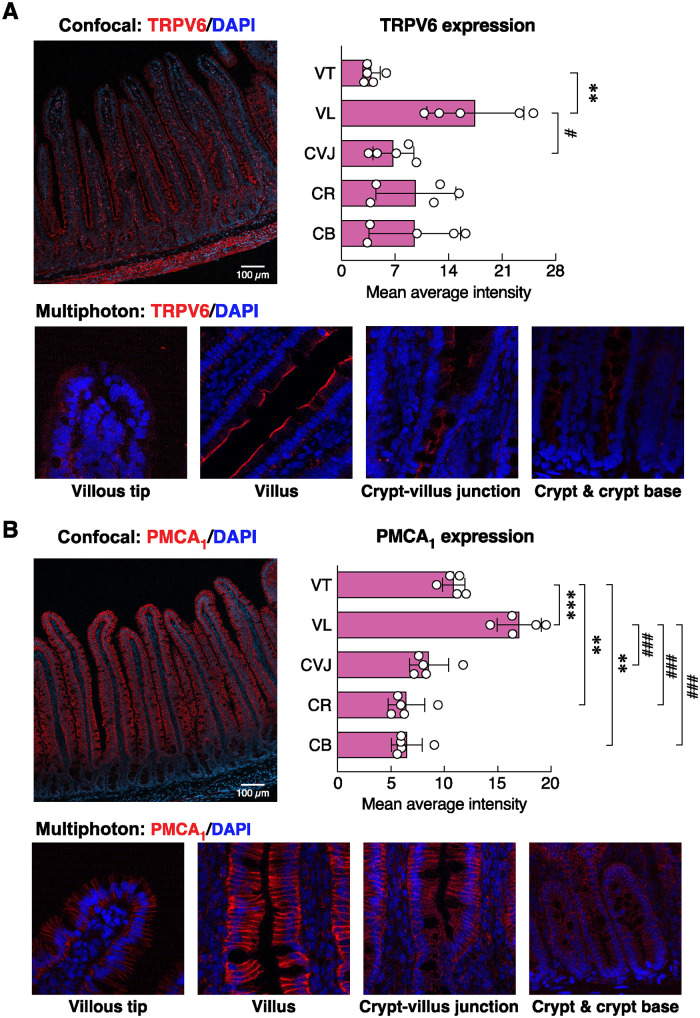
Expression of calcium transporter protein expressions along the crypt-villus axis in rat duodenum. Representative fluorescent photomicrographs of (A) TRPV6 and (B) PMCA_1_ distribution along different parts of the duodenal villus in 22-week-old rats as captured by a confocal laser-scanning microscope and a multiphoton microscope, i.e., villous tip (VT), villus (VL), crypt-villus junction (CVJ), crypt (CR), crypt base (CB). Red fluorescent signals represent TRPV6 and PMCA_1_ protein expression. Blue fluorescent signals represent nuclei. **P < 0.01, ***P < 0.001 vs. expression level at VT; ^#^*P* < 0.05, ^###^*P* < 0.001 vs. expression level at VL (n = 5; one-way ANOVA with Tukey’s multiple comparisons test).

**Fig 9:**
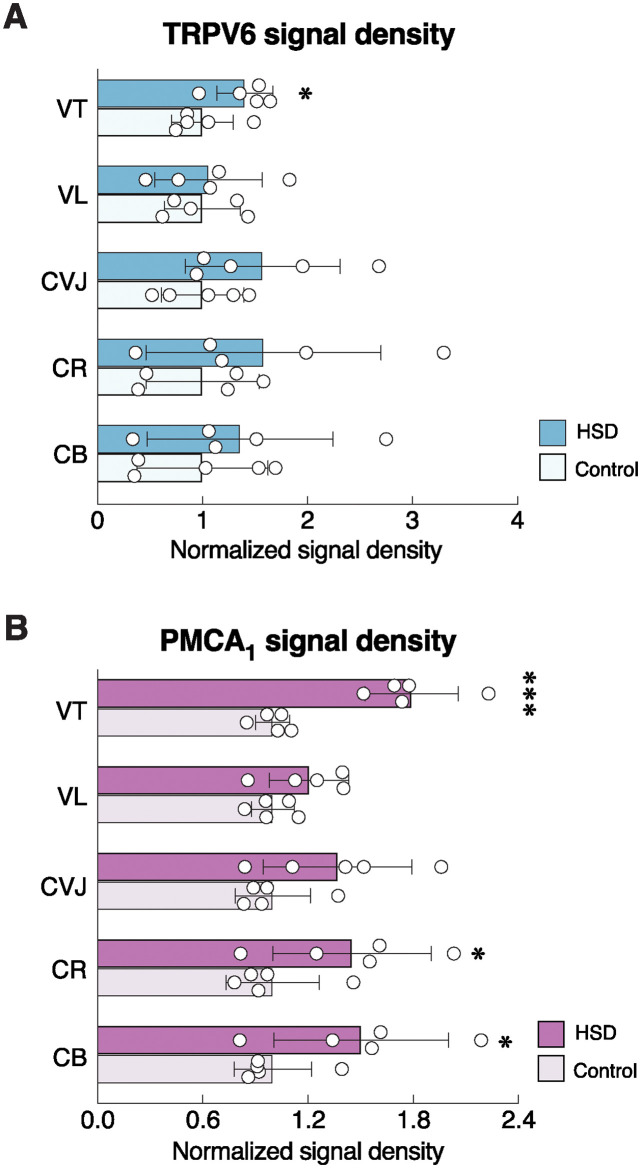
Effects of 3-month high-salt diet (HSD) ingestion on calcium transporter protein expressions along different parts of the duodenal villus. Normalized signal density of (A) TRPV6 and (B) PMCA_1_ expressions along different parts of the duodenal villus [i.e., villous tip (VT), villus (VL), crypt-villus junction (CVJ), crypt (CR), crypt base (CB)] in rats fed HSD for 3 months. Expression levels of age-matched control groups are normalized to 1. TRPV6, transient receptor potential vanilloid subfamily member 6; PMCA_1_, plasma membrane Ca^2+^-ATPase 1. (n = 5/group; unpaired Student’s *t*-test; *P < 0.05, ***P < 0.001 vs. age-matched control group).

As depicted in Figs 6–9, we also investigated distribution and localization of UGN, sodium transporter (i.e., NKA) and calcium transporters (i.e., TRPV6 and PMCA_1_) in the rat duodenum by using multiphoton and confocal microscopes. Red signals of UGN-producing cells were present in certain villous epithelial cells (Fig 6A). Although the UGN signal density in individual UGN-positive cells did not change (Fig 6C), the ratio of UGN-positive cells per total cells was significantly greater in HSD group than control group (Fig 6B), thus suggesting that HSD increased the number of UGN-producing cells. Moreover, NKA was found to be abundantly expressed in most duodenal epithelial cells especially at the basolateral membrane and exhibited differential crypt-villus distribution (Fig 7A). Specifically, NKA protein levels were most abundant in the villous area, and gradually declined in the crypt (Fig 7B). However, HSD did not alter NKA signal density in either villous or crypt areas (Fig 7C).

Since HSD has been reported to impair calcium and bone metabolism [6, 7, 37], consistent with altered levels of bone resorption (CTX1) and formation markers (P1NP) in the present study (S1 Fig), we further investigated the protein expression and localization of salient intestinal calcium transporters, i.e., TRPV6 and PMCA_1_, in HSD vs. ND control groups. Positive red signals of TRPV6 were predominantly localized in the apical membrane—consistent with its physiological role for calcium uptake [38]—and some signals also dispersed inside the cytoplasm of epithelial cells (Fig 8A). Differential crypt-villus expression analysis revealed that TRPV6 protein levels were apparently highest at the villous region, while the lowest expression was found at the villous tip (Fig 8A), suggesting that the highest calcium absorption rate occurred at the villous region rather than the villous tip. Nevertheless, HSD enhanced TRPV6 signal density only at the villous tip, but not the villous or crypt areas (Fig 9A). PMCA_1_ was abundantly expressed in the basolateral membrane of duodenal epithelial cells (Fig 8B). Similar to TRPV6 expression, PMCA_1_ was localized mostly at the villous region and gradually declined in the crypt—i.e., villous > villous tip > crypt-to-villous junction ≈ crypt ≈ crypt base (Fig 8B). HSD was able to enhance PMCA_1_ signal density in both villous and crypt areas, particularly at the villous tip, crypt, and crypt base (Fig 9B).

## Discussion

**Fig 10:**
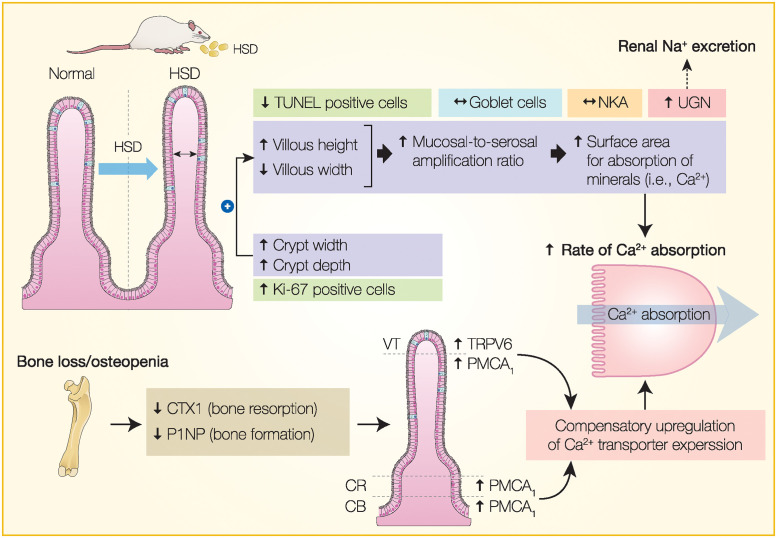
A diagram summarizes how high-salt diet (HSD) altered the intestinal morphology and the expression of calcium transporters, namely TRPV6 and PMCA_1_. It is likely that an increase in the intestinal absorptive surface area, together with the unregulated TRPV6 and PMCA_1_ expression, helped enhance the rate of Ca^2+^ absorption as a compensatory mechanism to counterbalance HSD-induced bone loss. Indeed, HSD probably suppressed both bone formation and resorption as indicated by decreased P1NP and CTX1 levels, respectively. Meanwhile, HSD led to greater UGN expression, which might enhance renal Na^+^ excretion. An arrow with a dashed line indicates a hypothetical mechanism. CB, crypt base; CR, crypt; CTX1, C-terminal telopeptide of type 1 collagen; NKA, Na^+^/K^+^-ATPase; P1NP, N-terminal propeptide of type 1 procollagen; PMCA_1_, plasma membrane Ca^2+^-ATPase 1; TRPV6, transient receptor potential vanilloid subfamily member 6; UGN, uroguanylin; VT, villous tip.

Previously, high-salt intake was demonstrated to cause a number of detrimental effects on bone metabolism in rats by increasing cortical and trabecular bone porosity, leading to impaired bone mechanical properties [6]. Consistent with HSD-induced osteopathy, the levels of bone remodeling biomarkers, namely CTX1 and P1NP, were reduced in HSD rats (S1 Fig). Decreases in serum CTX1 and P1NP levels suggested a reduction in bone turnover, where both bone resorption and formation inappropriately declined. Decreased bone resorption without compensatory formation led to the accumulation of bone microdamage and porosity. Since several proxy indicators of bone strength, e.g., yield load and ultimate displacement, were observed only in short-term HSD treatment (1 month) but not in long-term treatment [6], we postulated that certain compensatory mechanisms occurred, helping to counterbalance the negative effects of HSD on bone, presumably by providing more calcium as a precursor for mineralization. Thus, the intestinal morphological changes and upregulation of calcium transporter protein expression—i.e., TRPV6 and PMCA_1_—as demonstrated in the present study could be important adaptations during high-salt intake (Fig 10).

Under normal conditions, exogenous calcium was solely taken up into the body via the alimentary tract (for review, please see [39–41]. The absorptive rates of calcium and several other minerals (e.g., magnesium) were positively dependent on various factors, especially mucosal surface area, and abundance of transporting proteins [42]. On the other hand, an increase in the thickness of mucous layer covering the mucosal surface could hinder ion diffusion [43–45]. The present histomorphometric study revealed that the villous height and crypt depth were markedly greater in HSD rats compared to age-matched control rats (Fig 2), thereby resulting in a greater mucosal-to-serosal amplification ratio. Such an increase in the absorptive surface area appeared to be a compensatory mechanism to help increase calcium uptake. The underlying cellular mechanism of their increased surface area was elusive, but it was likely to be due to proliferation of the intestinal stem cells in the crypt together with a decrease in apoptosis of mature enterocytes in the villous area. Generally, a balance between proliferation and apoptosis is crucial for calcium absorption, as calcium transport across the epithelium requires cellular calcium transport machinery, e.g., TRPV6, calbindin-D_9k_, and tight junction proteins [24, 41]. Excessive apoptosis of villous enterocytes reduces absorptive surface area [46]. Conversely, while increased enterocyte proliferation may increase calcium absorption, there is a risk of disorganized tissue architecture, which can reduce the efficiency of calcium or other nutrient absorption if the newly proliferated cells are immature or not properly differentiated [9].

In agreeing with our hypothesis, HSD was able to upregulate TRPV6 and PMCA_1_ protein expression (Fig 9), particularly in the villous region where transcellular calcium uptake was predominant [47]. The cellular and molecular mechanisms undergoing the high-salt intake-induced increases in calcium transporter expression remained unclear. But since the mRNA expression of both transporters was reportedly unaltered in HSD rats [6], the compensatory mechanisms must have occurred at the translational rather than transcriptional levels. In addition, the upregulated PMCA_1_ protein expression observed in the crypt region of HSD rats (Fig 9B) might, in turn, reflect increases in the proliferation and differentiation of crypt cells [48].

Meanwhile, there were several HSD-associated adaptations that probably helped prevent excessive Na^+^ retention in the body (Fig 1E), which could indirectly alleviate the negative effects of high-salt diet on bone remodeling. Although fecal Na^+^ output was relatively constant throughout the 4-month treatment (Fig 1B), urinary Na^+^ output escalated up to ~800 mg/day (Fig 1C), implying that certain factors especially enhanced renal Na^+^ excretion and helped maintain serum Na^+^ levels at 5 months (Fig 1F). Among several natriuretic peptides for urinary sodium excretion, UGN has been recognized as an intestine-derived peptide with natriuretic activity [18, 49]. Specifically, intestinal mucosal cells are able to sense and respond to dietary salt by secreting UGN as an endocrine factor, which, in turn, activates the guanylyl cyclase-C receptor on the apical membrane of renal tubular cells. This activation increases intracellular cyclic GMP (cGMP) levels in order to inhibit NHE3 and epithelial sodium channels (ENaC) in the tubular cells. Consequently, this reduces sodium reabsorption and enhances urinary sodium excretion [17, 19]. Enteral sodium chloride loading in UGN-knockout mice showed a reduction in sodium excretion compared with wild-type mice [50]. Hence, UGN is likely a humoral factor that fine-tunes urinary sodium excretion to match oral sodium consumption, thereby preventing excessive salt retention. In other words, like many other anticipatory responses (e.g., intestinal L-cell–pancreatic β-cell communication), this gut-kidney axis tightly coordinated dietary salt intake with natriuresis and helped prevent abrupt changes in serum Na^+^ levels and osmolarity [51]. Interestingly, HSD seemed to induce an increase in the number of UGN-positive cells in the mucosal area exposed to high Na^+^ concentration rather than upregulating the expression level of UGN (Fig 6). However, future experiments are required to demonstrate the underlying mechanisms of HSD-induced UGN-positive precursor cell proliferation and how this endocrine factor modulates renal Na^+^ excretion during high-salt intake.

Besides UGN production, other compensatory or protective mechanisms may involve the mucous barrier and unstirred water layer, the latter of which defined the diffusion barrier formed by a thin layer of fluid or mucin-rich materials (~100–500 μm thick) close to the villous surface and deep into the crypt [52]. Under normal conditions, the mucus barrier and unstirred water layer were able to reduce fluxes of ions, including Ca^2+^, Na^+^ and several other small molecules [45, 53]. They generally determine ion exchange between the central lumen and the fluid layer close to the mucosal surface, thereby affecting favorable conditions (e.g., concentration gradient) for ion transport across the epithelium [43]. Formation of the mucus layer depends on the number of goblet cells in the mucosal epithelium [20, 54]. However, in the present study, we did not observe a significant change in the percentage of goblet cells relative to total epithelial cells (Fig 5), suggesting that goblet cell production was unlikely to be a compensatory response during HSD treatment. Whether goblet cells indeed enhanced mucin secretion remains unknown and is worth exploring in future experiments. Moreover, there is evidence that high salt consumption results in notable alterations of the intestinal microbiome. Since metabolites produced by microbiota (e.g., butyrate and propionate) can enhance intestinal calcium transport [55–57], prolonged high-salt intake likely causes microbiome dysbiosis, which may impair intestinal calcium absorption.

In conclusion, we have elaborated on the morphological adaptations and the upregulation of key calcium transporter proteins (i.e., TRPV6 and PMCA_1_) in the intestine of HSD-treated rats. Since long-term high-salt consumption is known to cause osteopenia and impair calcium metabolism, increases in the mucosal surface area and calcium transporters likely helped enhance dietary calcium uptake to mitigate the negative calcium balance (although it was not fully restored). Meanwhile, after exposure to high luminal sodium content, some epithelial cells became UGN-positive, and possibly released UGN as an endocrine natriuretic peptide, signaling renal tubular cells to enhance urinary Na^+^ excretion. Although future experiments are required to elucidate the underlying molecular mechanisms, our findings shed light on the existence of pathophysiological adaptations in the rat small intestine, which may represent another target organ for drug discovery against HSD-induced osteopathy. For instance, since high intestinal calcium absorption capacity persists under HSD conditions, oral calcium supplementation is probably beneficial for this group of patients.

## Supporting information

S1 Fig Effects of high-salt diet (HSD) on the serum levels of bone turnover markers. Serum levels of (A) bone resorption marker CTX-1 and (B) bone formation marker P1NP in rats fed HSD for 1–4 months as determined by ELISA. CTX-1, C-terminal telopeptide of type 1 collagen; P1NP, N-terminal propeptide of type 1 procollagen (n = 8/group; unpaired Student’s *t*-test; *P < 0.05, **P < 0.01 vs. age-matched control group).
(PDF)

S1 Table Two-way ANOVA analysis of percent Na^+^ retention. Two-way ANOVA table shows interaction between high-salt diet (HSD) treatment and duration of treatment on percent Na^+^ retention.
(PDF)

S1 File Raw data of all experiments. The table shows raw data obtained from high-salt diet (HSD) fed and age-matched control groups from all experiments, i.e., body sodium balance; intestinal histomorphometric analysis; goblet cell staining; determination of Ki-67-positive and TUNEL-positive cells by immunofluorescent technique, immunolocalization of uroguanylin, NKA and calcium transport-related proteins in the intestine are determined by immunofluorescent technique. Serum bone resorption marker CTX-1 and bone formation marker P1NP obtained from high-salt diet (HSD) fed and age-matched control groups are determined.
(PDF)
