# Supplementary figures and images for: Long-term excessive salt consumption alters villous and crypt morphology and the protein expression of uroguanylin, TRPV6 and PMCA1b in the rat small intestine
Source: PLoS One. 2025 Jan 16;20(1):e0317415. doi: 10.1371/journal.pone.0317415 (PMC11737712; doi:10.1371/journal.pone.0317415)

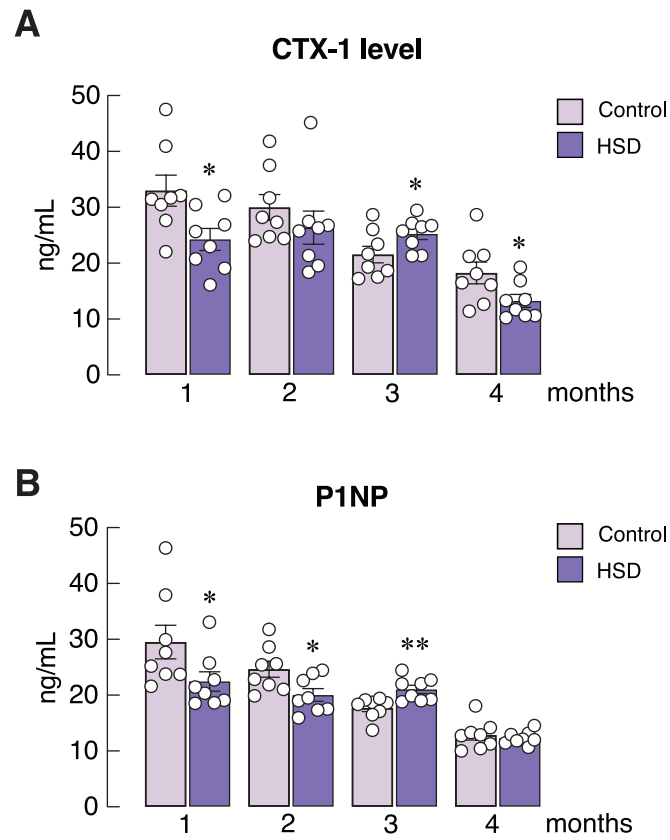

**Supplementary Figure S1:** Thonapan et al.

Supplement: S1 Fig — Serum levels of (A) bone resorption marker CTX-1 and (B) bone formation marker P1NP in rats fed HSD for 1–4 months as determined by ELISA. CTX-1, C-terminal telopeptide of type 1 collagen; P1NP, N-terminal propeptide of type 1 procollagen (n = 8/group; unpaired Student’s t-test; *P < 0.05, **P < 0.01 vs. age-matched control group). (PDF) [file pone.0317415.s001.pdf]
