# Supplementary material for: Long-term excessive salt consumption alters villous and crypt morphology and the protein expression of uroguanylin, TRPV6 and PMCA1b in the rat small intestine
Source: PLoS One. 2025 Jan 16;20(1):e0317415. doi: 10.1371/journal.pone.0317415 (PMC11737712; doi:10.1371/journal.pone.0317415)
Supplement: S1 Table — Two-way ANOVA table shows interaction between high-salt diet (HSD) treatment and duration of treatment on percent Na+ retention. (PDF) [file pone.0317415.s002.pdf]

Supplementary Table S1

Two-way ANOVA analysis of percent Na^+ retention

Parameter	Sum of Squares	DF	Mean Square	F value	P value
**HSD treatment**	19880	1	19880	1024	P<0.0001
**Duration of treatment**	24392	3	8131	418.9	P<0.0001
**HSD treatment × Duration of treatment (Interaction)**	14011	3	4670	240.6	P<0.0001
**Error**	815.2	42	19.41		
