# Supplementary material for: Long-term excessive salt consumption alters villous and crypt morphology and the protein expression of uroguanylin, TRPV6 and PMCA1b in the rat small intestine
Source: PLoS One. 2025 Jan 16;20(1):e0317415. doi: 10.1371/journal.pone.0317415 (PMC11737712; doi:10.1371/journal.pone.0317415)
Supplement: S1 File — The table shows raw data obtained from high-salt diet (HSD) fed and age-matched control groups from all experiments, i.e., body sodium balance; intestinal histomorphometric analysis; goblet cell staining; determination of Ki-67-positive and TUNEL-positive cells by immunofluorescent technique, immunolocalization of uroguanylin, NKA and calcium transport-related proteins in the intestine are determined by immunofluorescent technique. Serum bone resorption marker CTX-1 and bone formation marker P1NP obtained from high-salt diet (HSD) fed and age-matched control groups are determined. (PDF) [file pone.0317415.s003.pdf]

Raw data for Figure 1

Fig 1A. Daily sodium intake (mg)

	Daily sodium intake (mg)							
	1 month		2 months		3 months		4 months	
N	Control	HSD	Control	HSD	Control	HSD	Control	HSD
1	259.996	852.595	262.789	786.338	285.899	795.939	273.463	867.545
2	261.658	738.81	259.198	845.513	282.873	851.729	294.877	890.837
3	265.682	764.384	260.461	868.568	279.149	892.49	278.151	958.431
4	264.219	799.952	222.92	890.68	258.998	785.001	275.99	920.975
5	283.039	802.863	233.793	831.034	273.197	907.362	280.246	866.995
6	278.251	824.896	270.736	794.05	299.266	919.008	-	-
7	-	-	-	-	289.224	815.926	-	-
8	-	-	-	-	289.523	796.096	-	-
Mean	268.808	797.25	251.65	836.031	282.266	845.444	280.545	900.957
SEM	3.8786	16.6948	7.67514	16.7251	4.30327	19.3229	3.75586	17.4301

Fig 1B. Fecal sodium excretion (mg)

	Fecal sodium excretion (mg)							
	1 month		2 months		3 months		4 months	
N	Control	HSD	Control	HSD	Control	HSD	Control	HSD
1	8.24808	38.0803	5.73031	42.5523	4.90193	37.0364	5.46473	35.0206
2	3.18109	39.3817	8.1003	44.775	8.19516	49.8647	4.74777	34.8428
3	7.6033	43.4995	6.15947	80.0895	5.9261	49.641	4.70852	40.7517
4	5.15443	41.0853	6.83305	48.8465	3.38561	59.8919	6.51345	27.9975
5	8.12891	44.7777	5.39563	72.2471	5.19593	47.0746	5.85404	39.1316
6	9.98468	34.3926	6.21594	66.7108	3.76347	50.1681	-	-
7	-	-	-	-	4.27575	41.1408	-	-
8	-	-	-	-	5.57274	48.3511	-	-
Mean	7.05008	40.2029	6.40578	59.2035	5.15209	47.8961	5.4577	35.5489
SEM	1.00133	1.54396	0.39274	6.46898	0.53314	2.39207	0.34184	2.21027

Fig 1C. Urinary sodium excretion (mg)

	Urinary sodium excretion (mg)							
	1 month		2 months		3 months		4 months	
N	Control	HSD	Control	HSD	Control	HSD	Control	HSD
1	0.93944	18.7189	1.06778	30.9875	28.0938	773.524	39.2778	817.931
2	0.98889	19.6167	1.17403	30.8368	35.1389	577.83	47.7917	775.583
3	0.97708	17.2825	0.53	36.6975	30.625	600.139	32.4444	775.323
4	1.12542	18.3021	0.6775	32.1071	39	770.833	44.9167	861.278
5	1.05875	20.9283	0.76528	34.2925	34.2222	618.556	34.4167	854.778
6	1.25417	17.3506	0.64694	34.7304	32.2778	746.611	-	-
7	-	-	-	-	33.8611	741.319	-	-
8	-	-	-	-	34.4722	553.469	-	-
Mean	1.05729	18.6999	0.81025	33.2753	33.4614	672.785	39.7694	816.978
SEM	0.04775	0.57139	0.10384	0.95583	1.14571	33.107	2.94448	18.4944

Fig 1D. Total sodium excretion (mg)

	Total sodium excretion (mg)							
	1 month		2 months		3 months		4 months	
N	Control	HSD	Control	HSD	Control	HSD	Control	HSD
1	9.18752	53.5991	6.90434	73.219	36.2889	614.866	42.6357	838.102
2	6.30234	58.9984	5.12799	76.8821	47.3824	660.031	53.2564	838.511
3	8.58038	59.019	6.83697	109.903	36.5511	604.852	54.8403	884.363
4	6.56249	59.3873	7.62055	83.5769	42.3856	791.784	40.412	897.018
5	9.38307	50.3099	7.05313	93.9529	39.4182	659.696	43.6882	874.214
6	13.2347	50.8154	6.86288	87.5641	36.0412	771.169	-	-
7	-	-	-	-	38.1369	772.807	-	-
8	-	-	-	-	40.045	601.82	-	-
Mean	8.87508	55.3548	6.73431	87.5163	39.5312	684.628	46.9665	866.442
SEM	1.02332	1.75218	0.3427	5.40249	1.36004	28.6799	2.94979	12.0411

Fig 1E. Percent of sodium retention

	Percent of sodium retention							
	1 month		2 months		3 months		4 months	
N	Control	HSD	Control	HSD	Control	HSD	Control	HSD
1	97.0506	91.0434	97.6142	91.3325	88.3888	19.9233	86.6288	3.39385
2	97.4631	90.5105	97.1153	90.907	88.2094	26.0461	85.2504	4.63757
3	96.7208	92.2789	97.6584	87.6608	85.8875	22.9488	85.6302	0.88977
4	97.1754	92.5761	97.492	89.943	84.4853	39.3807	86.34	5.87383
5	97.3474	92.652	96.9832	87.9299	83.8777	13.8436	86.4968	0.79584
6	95.2436	92.9914	97.4651	87.2897	86.3711	19.1475	-	-
7	-	-	-	-	88.1179	2.92535	-	-
8	-	-	-	-	86.1687	20.8928	-	-
Mean	96.8335	92.0087	97.388	89.1772	86.4383	20.6385	86.0692	3.11817
SEM	0.33491	0.40624	0.11245	0.72209	0.60524	3.65615	0.26762	1.0084

Fig 1F. Serum sodium (mmol/L)

	Serum sodium (mmol/L)			
	1 month		5 months	
N	Control	HSD	Control	HSD
1	142	146	145	146
2	144	146	146	145
3	142	148	145	143
4	142	145	146	146
5	141	142	146	146
6	141	146	145	144
Mean	142	145.5	145.5	145
SEM	0.44721	0.80623	0.22361	0.5164

Fig 1G. Serum osmolality (mOsm/kg)

	Serum osmolality (mOsm/kg)			
	1 month		5 months	
N	Control	HSD	Control	HSD
1	310	315.5	319	324.5
2	316	318	319.5	325.5
3	318.5	311.5	321	332.5
4	314.5	316	321	330
5	311	315.5	319	333.5
6	309	323.5	323	324
Mean	313.167	316.667	320.417	328.333
SEM	1.53116	1.61589	0.63792	1.71594

Raw data for Figure 2

Fig 2A. Villous height (μm)

	Villous height (μm)							
	1 month		2 months		3 months		4 months	
N	Control	HSD	Control	HSD	Control	HSD	Control	HSD
1	571.014	609.881	506.975	585.29	471.677	459.53	519	600.186
2	560.859	606.279	524.187	650.967	535.782	520.141	571.044	581.878
3	519.361	626.863	505.052	627.43	526.52	573.805	520.804	632.968
4	574.894	603.468	590.745	614.969	454.155	599.838	522.028	589.664
5	554.791	573.239	553.633	548.151	520.523	484.669	493.421	573.601
6	445.437	573.921	580.056	588.059	489.488	510.945	-	-
7	-	-	-	-	532.023	568.528	-	-
8	-	-	-	-	597.46	593.81	-	-
Mean	537.726	598.942	543.441	602.478	515.953	538.908	525.259	595.659
SEM	20.1415	8.68002	15.1177	14.815	15.7589	18.5157	12.6083	10.3076

Fig 2B. Villous width (μm)

	Villous width (μm)							
	1 month		2 months		3 months		4 months	
N	Control	HSD	Control	HSD	Control	HSD	Control	HSD
1	107.385	104.016	105.469	110.847	76.0285	89.1408	111.476	106.534
2	104.11	91.337	102.076	103.905	78.5682	82.7126	103.658	101.849
3	110.847	102.573	101.016	96.9563	77.9793	75.2761	110.618	98.156
4	96.818	100.955	107.266	104.713	78.939	99.0503	110.455	100.561
5	102.975	101.542	103.464	105.341	94.7286	95.518	103.028	102.525
6	119.385	97.429	100.273	100.015	86.182	89.8631	-	-
7	-	-	-	-	78.9355	91.5558	-	-
8	-	-	-	-	89.377	87.0855	-	-
Mean	106.92	99.642	103.261	103.629	82.5922	88.7753	107.847	101.925
SEM	3.1439	1.8879	1.10054	1.94822	2.36599	2.61153	1.84966	1.37247

Fig 2C. Crypt depth (μm)

	Crypt depth (μm)							
	1 month		2 months		3 months		4 months	
N	Control	HSD	Control	HSD	Control	HSD	Control	HSD
1	102.793	95.4518	103.884	110.359	112.36	120.022	96.6915	98.0378
2	133.551	115.753	116.756	95.8378	138.357	135.598	92.694	108.694
3	105.072	108.435	94.612	108.57	103.321	129.362	112.588	107.819
4	108.32	100.049	122.221	96.7165	117.235	149.002	106.693	109.627
5	98.661	80.389	121.714	101.341	126.426	146.98	84.9783	127.507
6	111.311	107.6	98.3323	111.915	122.872	130.23	-	-
7	-	-	-	-	129.515	148.674	-	-
8	-	-	-	-	125.154	108.551	-	-
Mean	109.951	101.28	109.587	104.123	121.905	133.552	98.7289	110.337
SEM	5.04632	5.07454	4.97166	2.89035	3.82372	5.15578	4.92515	4.77301

Fig 2D. Crypt width (μm)

	Crypt width (μm)							
	1 month		2 months		3 months		4 months	
N	Control	HSD	Control	HSD	Control	HSD	Control	HSD
1	44.9258	41.9298	42.6323	43.6883	31.7859	43.6248	45.3403	47.6938
2	42.7565	35.5348	36.193	48.2563	32.2248	38.582	45.4815	46.5954
3	40.7503	43.576	38.5153	48.5158	37.311	38.7093	50.1603	47.2375
4	42.3418	37.6532	44.2288	43.1913	34.5315	47.4363	41.9113	44.9055
5	43.0113	45.086	37.6098	47.0345	40.1685	38.0243	43.2115	40.491
6	43.9175	37.775	42.1245	45.6565	36.3848	41.4413	-	-
7	-	-	-	-	31.3179	40.3683	-	-
8	-	-	-	-	40.5723	38.9433	-	-
Mean	42.9505	40.2591	40.2173	46.0571	35.5371	40.8912	45.221	45.3846
SEM	0.57993	1.5532	1.30961	0.92775	1.2992	1.14169	1.40483	1.31164

Fig 2E. Villus-to-crypt ratio

	Villus-to-crypt ratio							
	1 month		2 months		3 months		4 months	
N	Control	HSD	Control	HSD	Control	HSD	Control	HSD
1	5.555	6.38941	4.88019	5.30351	4.19791	3.82873	5.36758	6.12199
2	4.1996	5.23768	4.48959	6.79238	3.87248	3.83592	6.16052	5.35336
3	4.94292	5.78103	5.33814	5.77904	5.09596	4.43567	4.62576	5.87066
4	5.30737	6.03175	4.83341	6.35847	3.87389	4.02571	4.89282	5.37885
5	5.6232	7.13081	4.54863	5.40896	4.11723	3.29753	5.80643	4.49857
6	4.00173	5.33382	5.89893	5.2545	3.98373	3.9234	-	-
7	-	-	-	-	4.10783	3.824	-	-
8	-	-	-	-	4.77382	5.47035	-	-
Mean	4.9383	5.98408	4.99815	5.81614	4.25285	4.08016	5.37062	5.44468
SEM	0.28334	0.28883	0.21823	0.25772	0.15729	0.22708	0.28258	0.27821

Fig 2F. Mucosal-to-serosal amplification ratio

	Mucosal-to-serosal amplification ratio							
	1 month		2 months		3 months		4 months	
N	Control	HSD	Control	HSD	Control	HSD	Control	HSD
1	11.0757	12.4051	10.244	11.3523	12.8431	9.84486	9.90546	11.2754
2	11.3258	14.2427	11.6499	12.2192	14.2145	12.2319	11.1619	11.287
3	10.4854	12.5487	10.9579	12.0543	12.8983	13.8618	9.4413	12.3005
4	12.0128	13.1537	11.5427	12.2736	11.6536	11.6181	10.4093	11.7312
5	11.225	11.3499	12.1031	10.4699	11.3456	10.8721	10.012	11.987
6	8.44199	12.7162	11.978	11.6152	11.738	11.1843	-	-
7	-	-	-	-	14.3065	12.4825	-	-
8	-	-	-	-	13.1757	13.5456	-	-
Mean	10.7611	12.7361	11.4126	11.6641	12.7719	11.9551	10.186	11.7162
SEM	0.5051	0.38777	0.28532	0.28033	0.40032	0.47909	0.28861	0.19918

Raw data for Figure 3

Fig 3B.

Number of Ki-67-positive cells/crypt

	Number of Ki-67-positive cells/crypt							
	1 month		2 months		3 months		4 months	
N	Control	HSD	Control	HSD	Control	HSD	Control	HSD
1	2.5	1.66667	3.4	2.5	3.3	2.88889	2.45455	2.90909
2	1.7	3	2.8	4.2	2.1	7.07692	1	3.5
3	2.1	3	3	2.58333	2.4	3.83333	1.9	5.9
4	2.6	2.8	1.875	2	2.54546	6.63636	2.27273	5.2
5	2.45455	2	3.18182	4.38462	2.22222	4.90909	0.71429	4.2
Mean	2.27091	2.49333	2.85136	3.13359	2.51354	5.06892	1.66831	4.34182
SEM	0.16578	0.27697	0.26346	0.48433	0.21075	0.7998	0.34597	0.54525

Percentage of Ki-67-positive cells/crypt

	Percentage of Ki-67-positive cells/crypt							
	1 month		2 months		3 months		4 months	
N	Control	HSD	Control	HSD	Control	HSD	Control	HSD
1	5.04267	2.89704	5.57637	4.88721	5.82763	4.74183	5.15445	5.5336
2	3.68511	5.43113	4.81857	7.86815	4.2241	12.889	2.06155	6.67605
3	3.7758	5.64358	5.08137	5.45326	4.43186	7.06611	3.33927	10.8957
4	5.34184	5.36138	3.63268	3.64212	4.76507	10.8559	4.31763	8.93105
5	4.76769	3.54001	6.29231	7.72283	3.78748	8.05059	1.38762	7.63564
Mean	4.52262	4.57463	5.08026	5.91472	4.60723	8.72068	3.2521	7.9344
SEM	0.33621	0.5648	0.44041	0.82215	0.34379	1.43094	0.69482	0.92734

Raw data for Figure 4

Fig 4B. Number of TUNEL-positive nuclei/villus

	Number of TUNEL-positive nuclei/villus							
	1 month		2 months		3 months		4 months	
N	Control	HSD	Control	HSD	Control	HSD	Control	HSD
1	10.7	1.88889	6.3	0.7	6.4	0	9.55556	1.28571
2	12.2222	1.2	4.1	1.9	27	3.9	15.1	6
3	12.9	0.7	3.2	0.2	12.9	0	14.4	2.6
4	3.7	0.6	5.4	3.8	13.1	9.7	20.3	0
5	1.5	4.1	9.3	0.1	9.3	0	36.4	0
Mean	8.20444	1.69778	5.66	1.34	13.74	2.72	19.1511	1.97714
SEM	2.34156	0.64248	1.05385	0.69325	3.53929	1.90142	4.63629	1.11507

Fig 4C. Percentage of TUNEL-positive nuclei/villus

	Percentage of TUNEL-positive nuclei/villus							
	1 month		2 months		3 months		4 months	
N	Control	HSD	Control	HSD	Control	HSD	Control	HSD
1	5.67795	0.92901	3.6964	0.37534	3.6214	0	5.69498	0.65021
2	6.66512	0.61812	2.34906	0.86051	17.3651	2.37637	6.99777	2.97262
3	6.30204	0.39895	1.71672	0.12307	7.89306	0	6.30431	1.27748
4	1.96684	0.28812	2.80577	2.39416	7.73625	6.23914	9.88669	0
5	0.86483	2.3925	4.95293	0.04673	6.12226	0	21.596	0
Mean	4.29536	0.92534	3.10418	0.75996	8.54762	1.7231	10.096	0.98006
SEM	1.19885	0.38276	0.5636	0.43261	2.33436	1.21919	2.96344	0.55175

Raw data for Figure 5

Fig 5B. Percentage of goblet cells/villus

	Percentage of goblet cells/villus							
	1 month		2 months		3 months		4 months	
N	Control	HSD	Control	HSD	Control	HSD	Control	HSD
1	20.5126	19.1205	20.7356	21.2544	20.4136	18.2582	21.3248	18.7897
2	21.2202	19.1067	19.2443	19.081	20.2203	19.2389	14.8755	19.6898
3	17.6414	18.8716	21.6288	18.7325	24.5247	22.3749	19.8012	18.229
4	20.9255	15.6444	19.0422	19.6804	19.124	16.7543	15.6179	22.2672
5	19.7268	22.0137	21.8558	19.717	23.0641	21.057	19.8122	22.2232
6	20.9762	17.4018	18.4954	22.1141	20.2007	18.8292	-	-
Mean	20.1671	18.6931	20.167	20.0966	21.2579	19.4187	18.2863	20.2398
SEM	0.54866	0.8636	0.58376	0.53604	0.84448	0.82156	1.2769	0.85126

Raw data for Figure 6

Fig 6B. Ratio of UGN-positive cells/total cells in rats fed HSD for 3 months

	Ratio of UGN-positive cells/total cells	
N	Control	HSD
1	0.04842998	0.060911684
2	0.044599753	0.050748099
3	0.058888043	0.087083498
4	0.044827973	0.070178487
5	0.04149468	0.050683313
Mean	0.047648086	0.063921016
SEM	0.003017099	0.006831819

Fig 6C. Fold changes of UGN signal density in rats fed HSD for 3 months

	Fold changes of UGN signal density	
N	Control	HSD
1	1.898971741	1.993623689
2	1.929056937	1.885239772
3	2.07801297	1.852373285
4	2.278083047	1.929341228
5	1.781294958	2.009615558
Mean	1.993083931	1.934038706
SEM	0.085512738	0.030277776

Raw data for Figure 7

Fig 7B. Mean average intensity of NKA protein expression from VT to CB in 22-week-old rat duodenum

	Mean average intensity of NKA protein expression in 22-week-old rat duodenum				
N	VT	VL	CVJ	CR	CB
1	46.685	58.9688	39.6425	26.765	19.51
2	38.6488	38.0163	27.59	17.1275	18.17
3	44.81	43.6975	27.605	16.5038	17.3213
4	31.9167	29.72	23.7267	16.705	16.0183
5	31.5667	30.2813	23.8886	17.3643	15.5367
6	36.49	36.4888	28.245	23.8833	23.4543
7	41.6471	39.2075	30.9388	25.81	23.89
8	41.9014	33.2729	29.6257	30.7938	29.7483
Mean	39.2082	38.7066	28.9078	21.8691	20.4561
SEM	1.98092	3.33376	1.77172	1.98853	1.72673

Fig 7C. Normalized signal density of NKA protein along different parts of the duodenal villus in rats fed HSD for 3 months.

	Normalized signal density of NKA protein									
	VT		VL		CVJ		CR		CB	
N	Control	HSD	Control	HSD	Control	HSD	Control	HSD	Control	HSD
1	1.1907	1.06139	1.52348	1.06067	1.37134	1.05266	0.95375	0.89619	0.95375	1.05527
2	0.98573	1.12904	0.98216	1.22757	0.95442	1.3957	0.88824	0.94723	0.88824	1.04193
3	1.14287	1.21295	1.12894	1.07672	0.95493	1.06368	0.84675	1.11985	0.84675	1.35595
4	0.81403	1.00668	0.76783	0.89759	0.82077	1.04457	0.78306	1.02148	0.78306	1.01895
5	0.8051	0.99979	0.78233	0.85237	0.82637	0.90707	0.75951	0.99429	0.75951	1.01168
6	0.93067	1.09099	0.9427	0.87437	0.97707	0.9433	1.14657	0.81269	1.14657	0.9291
7	1.06221	0.99848	1.01294	1.03603	1.07026	1.08298	1.16787	1.02617	1.16787	1.05513
8	1.06869	0.82483	0.85962	0.92288	1.02484	1.00756	1.45425	0.99684	1.45425	1.19492
Mean	1	1.04052	1	0.99352	1	1.06219	1	0.97684	1	1.08286
SEM	0.05052	0.04045	0.08613	0.04558	0.06129	0.05231	0.08441	0.03271	0.08441	0.04689

Raw data for Figure 8

Fig 8A. Mean average intensity of TRPV6 protein expression from VT to CB in 22-week-old rat duodenum

	Mean average intensity of TRPV6 protein expression in 22-week-old rat duodenum				
N	VT	VL	CVJ	CR	CB
1	5.835	25.0925	8.76875	15.37	16.2225
2	2.925	15.54	7.14625	12.8875	9.855
3	4.1325	23.2663	9.78	12.08	14.6925
4	3.3525	12.7725	4.65	4.53875	3.725
5	3.3475	10.825	3.515	3.76625	3.36125
Mean	3.9185	17.4993	6.772	9.7285	9.57125
SEM	0.51731	2.8429	1.18924	2.34326	2.67665

Fig 8B. Mean average intensity of PMCA1 protein expression from VT to CB in 22-week-old rat duodenum

	Mean average intensity of PMCA1 protein expression in 22-week-old rat duodenum				
N	VT	VL	CVJ	CR	CB
1	12.0475	14.295	8.2875	6.245	5.99125
2	11.1863	18.5938	11.755	9.40875	9.05875
3	9.2725	16.34	7.15125	5.0325	5.5975
4	10.5238	19.5288	8.025	5.9225	5.9175
5	11.4263	16.3913	7.6125	5.6425	5.95125
Mean	10.8913	17.0298	8.56625	6.45025	6.50325
SEM	0.47252	0.9234	0.82008	0.76602	0.64269

Raw data for Figure 9

Fig 9A. Normalized signal density of TRPV6 protein along different parts of the duodenal villus in rats fed HSD for 3 months.

	Normalized signal density of TRPV6 protein									
	VT		VL		CVJ		CR		CB	
N	Control	HSD	Control	HSD	Control	HSD	Control	HSD	Control	HSD
1	1.48909	1.51684	1.43392	1.07419	1.29485	1.95474	1.57989	1.98759	1.69492	1.51404
2	0.74646	1.64508	0.88804	1.83036	1.05526	2.68034	1.32472	3.29663	1.02965	2.74912
3	1.05461	1.35447	1.32956	1.15648	1.44418	1.26735	1.24171	1.07326	1.53507	1.12329
4	0.85556	0.96625	0.72989	0.45795	0.68665	0.94433	0.46654	0.36131	0.38919	0.3376
5	0.85428	1.53726	0.6186	0.7716	0.51905	1.01244	0.38714	1.18415	0.35118	1.06125
Mean	1	1.40398	1	1.05812	1	1.57184	1	1.58059	1	1.35706
SEM	0.13202	0.11889	0.16246	0.22898	0.17561	0.32976	0.24087	0.50058	0.27966	0.39648

Fig 9B. Normalized signal density of PMCA1 protein along different parts of the duodenal villus in rats fed HSD for 3 months.

	Normalized signal density of PMCA1 protein									
	VT		VL		CVJ		CR		CB	
N	Control	HSD	Control	HSD	Control	HSD	Control	HSD	Control	HSD
1	1.10616	1.73706	0.83941	0.85754	0.96746	0.83978	0.96818	0.81625	0.92127	0.81171
2	1.02709	2.23081	1.09184	1.39645	1.37225	1.41325	1.45866	1.60905	1.39296	1.61496
3	0.85137	1.6923	0.9595	1.25185	0.83482	1.51919	0.7802	1.55188	0.86072	1.56422
4	0.96626	1.77551	1.14674	1.40218	0.93682	1.96031	0.91818	2.03267	0.90993	2.18737
5	1.04912	1.51762	0.96251	1.12685	0.88866	1.11207	0.87477	1.24821	0.91512	1.33991
Mean	1	1.79066	1	1.20698	1	1.36892	1	1.45161	1	1.50363
SEM	0.04339	0.11854	0.05422	0.10113	0.09573	0.18977	0.11876	0.20218	0.09883	0.22239

Raw data for supplement Figure S1

Fig S1A. Serum levels of bone resorption marker CTX-1

	CTX-1 level (ng/ml)							
	1 month		2 months		3 months		4 months	
N	Control	HSD	Control	HSD	Control	HSD	Control	HSD
1	27.6396	32.103	37.5093	26.7908	17.5045	23.7355	18.1326	16.8888
2	47.5139	25.6938	41.8286	27.4965	19.3181	21.3606	28.66	11.7046
3	32.103	22.9853	28.2186	18.3981	21.6552	25.7613	16.4849	13.4752
4	22.0723	20.7797	31.6921	26.3061	18.6659	21.302	16.1358	13.2935
5	31.4481	19.0991	24.4394	21.3606	23.4207	27.1417	12.666	19.2632
6	30.4908	16.1358	24.7646	19.5385	28.66	29.4858	11.4049	10.647
7	31.6921	26.8607	27.3541	25.7613	26.0325	26.5129	21.2436	10.6054
8	40.9375	30.4908	23.9896	45.1643	17.2465	26.7211	21.4193	10.2338
Mean	32.9872	24.2685	29.9745	26.352	21.5629	25.2526	18.2684	13.2639
SEM	2.78075	1.96181	2.32913	2.95912	1.47806	1.0225	1.95031	1.155

Fig S1B. Serum levels of bone formation marker P1NP

	P1NP (ng/ml)							
	1 month		2 months		3 months		4 months	
N	Control	HSD	Control	HSD	Control	HSD	Control	HSD
1	21.5879	33.0733	25.4231	22.6021	18.6388	18.8274	14.1879	13.1667
2	46.371	18.5454	31.784	24.4853	18.7328	22.009	18.0564	12.126
3	29.9336	22.7642	25.6176	18.9862	19.1468	22.0478	12.7335	14.5438
4	23.7304	19.0824	28.6328	19.5059	13.7485	22.7235	11.2669	11.4625
5	27.6541	18.6388	22.8459	17.5241	17.1223	19.2439	10.4344	12.9486
6	25.231	20.3543	19.9074	17.3219	18.3602	19.8735	10.0343	11.8411
7	23.7739	21.4376	21.8545	15.9495	19.4071	18.9862	13.3212	10.657
8	37.9574	25.7652	21.0316	23.9048	16.484	24.4853	12.8837	12.0033
Mean	29.5299	22.4576	24.6371	20.035	17.7051	21.0246	12.8648	12.3436
SEM	3.00787	1.74475	1.43268	1.14259	0.66572	0.73628	0.90041	0.4209
